# Atrial depolarization abnormalities in pulmonary sarcoidosis

**DOI:** 10.1186/s43044-022-00312-7

**Published:** 2022-10-08

**Authors:** Amal Paul, Akhil Paul, Immanuel Subhash, Bijesh Yadav, John Roshan Jacob, D. J. Christopher, T. Balamugesh

**Affiliations:** 1 Department of Cardiology, Christian Medical College and Hospital (CMCH), Vellore, India; 2 Department of Pulmonary Medicine, Christian Medical College and Hospital (CMCH), Vellore, India; 3 Department of Biostatistics, Christian Medical College and Hospital (CMCH), Vellore, India; 4 Department of Cardiology and Cardiac Electrophysiology, Christian Medical College and Hospital (CMCH), Vellore, India; 5 MOSC Medical Mission Hospital, Aduputty Hills, Kunnamkulam, Thrissur, Kerala 680503 India

**Keywords:** Sarcoidosis, Electrocardiogram (ECG), Biphasic P, Cardiac sarcoidosis, Atrial depolarization

## Abstract

**Background:**

Cardiac sarcoidosis, often manifested as sudden death, can be the first manifestation of sarcoidosis. Since 12-lead electrocardiogram (ECG) is recommended as an initial screening tool for cardiac sarcoidosis, the recognition of subtle abnormalities assumes utmost significance. The objective of this study was to identify the electrocardiographic abnormalities in patients with pulmonary sarcoidosis.

**Results:**

A detailed analysis of 12-lead ECGs obtained from sixty patients with histopathologically proven pulmonary sarcoidosis and no overt cardiac involvement was done. The findings were compared with those of an age-matched control group. Varying degrees of intraventricular conduction defects were common in the study group [67%], as well as the control group [57%] [*P* = 0.23]. There was a higher prevalence of biphasic P wave [*P* = 0.003] and bifid P wave [*P* = 0.029] in lead III and rsr’ in lead aVF [*P* = 0.03] in the study group as compared to the control group.

**Conclusions:**

Our study demonstrates a greater prevalence of subtle ECG abnormalities in patients with pulmonary sarcoidosis as compared to patients with other forms of pulmonary disease. Atrial depolarization abnormalities were commoner in patients with pulmonary sarcoidosis.

**Supplementary Information:**

The online version contains supplementary material available at 10.1186/s43044-022-00312-7.

## Background

Sarcoidosis is a multisystem, granulomatous disease seen worldwide, with a reported prevalence of 4.7–64 in 100,000 [1–3]. It is believed to result from an immunological response to an unidentified antigenic trigger in individuals with genetic susceptibility [4]. More than 90% of patients have involvement of the lungs. The disease can also affect the skin, gastrointestinal tract, eyes, liver, heart, parotid gland, and spleen.

Myocardial involvement occurs in at least 25% of patients with sarcoidosis and accounts for as many as 13–25% of deaths from sarcoidosis [5]. An estimated 20–25% of patients with pulmonary/systemic sarcoidosis have asymptomatic cardiac involvement (clinically silent disease). About 40–50% of patients who die of sarcoidosis-related complications are found to have cardiac sarcoidosis at necropsy despite never having manifested any clinical evidence of myocardial involvement during their lifetime [6, 7]. The predominant sites of myocardial involvement, in decreasing order of frequency, are the left ventricular free wall and papillary muscles, the basal aspect of the ventricular septum, the right ventricular free wall, and the atrial walls [8]. Clinical picture of manifest cardiac sarcoidosis depends on the location, extent, and activity of the disease. The principal manifestations are conduction abnormalities, ventricular arrhythmias, and heart failure.

There is a growing realization that cardiac sarcoidosis could be the first manifestation of sarcoidosis in any organ. Cardiac sarcoidosis (CS) has been identified as the underlying aetiology in 16–35% of patients presenting with complete atrioventricular (AV) block (age < 60 years) [9, 10] or ventricular tachycardia (VT) of unknown aetiology [11, 12]. In patients with cardiac sarcoidosis, the predominant features reported in the literature include ventricular arrhythmias, bundle branch blocks, supraventricular arrhythmias, and sudden death [13]. The abnormalities in ECG may result from edema, granulomatous infiltration, and/or fibrosis associated with CS [14]. Atrial arrhythmias are believed to reflect atrial dilatation secondary to ventricular dysfunction, pulmonary parenchymal involvement, or direct atrial involvement from granulomas or scar tissue [15].

The expert consensus statement recommends screening for cardiac involvement with a 12-lead electrocardiogram in patients with biopsy-proven extra-cardiac sarcoidosis (class IIa), and advanced cardiac imaging [cardiac MRI (CMR) or positron emission tomography (PET)] in patients with one or more abnormalities detected on initial screening by symptoms, electrocardiogram [ECG], or echocardiogram (class IIa) [16, 17].

In view of the heavy weightage given for ECG as an initial screening tool in patients with extra-cardiac sarcoidosis, a thorough knowledge of subtle ECG abnormalities assumes utmost significance for the appropriate selection of patients for advanced screening. We conducted a detailed analysis of 12-lead ECGs from patients with histopathologically proven extra-cardiac sarcoidosis and compared the findings with those of a control group. The aim of the study was to identify the subtle ECG abnormalities common in this pathologic cohort, beyond what is attributable to the lung disease. Patients with manifest cardiac sarcoidosis were excluded so as to avoid skewing of the data with more advanced abnormalities such as complete atrioventricular block and ventricular tachyarrhythmias.

## Methods

This study was conducted in a tertiary care referral center among patients attending the pulmonology clinic, who were diagnosed to have pulmonary sarcoidosis. The study was approved by the institutional review board and the ethics committee. Patients who were known to have any form of cardiac sarcoidosis were not included in the study. All sixty patients included in the study group had histopathologic confirmation for the diagnosis of pulmonary sarcoidosis. No patient in this cohort had any indication for further evaluation for cardiac sarcoidosis as per existing recommendations [17].

Since lung disease can effect a myriad of changes in the cardiac physiology and potentially the surface ECG, our study included patients with other forms of pulmonary disease as an age-matched control group for comparative analysis of the ECG findings. The control group included 60 patients from the pulmonology clinic, mostly with mild forms of obstructive airway disease, and no known cardiac disease.

Analysis of 12-lead ECGs was done in all the patients included in the study, and findings documented. The findings were verified by another expert to account for inter-observer variability. The investigators analyzing the ECGs were blinded to the underlying pulmonary diagnosis. Details including demographic profile, co-morbidities, the Siltzbach stage of pulmonary sarcoidosis, duration of disease and treatment, and the profile of pulmonary function tests were documented by a separate investigator. Acquired data were summarized using mean with standard deviation for continuous variables and percentage for categorical variables. Continuous variables were compared using independent t test, and categorical variables with Chi-square test. Statistical analysis of the data was done using SPSS 25 software.

## Results

### Clinical profile

There were 60 patients with pulmonary sarcoidosis in the study group, of which majority [70%] were in stage 2 of Siltzbach classification system based on the appearance on chest X-ray. The mean duration of the disease was 35 ± 41 months [range: 2.5–182]. There were 31 patients [51.7%], who were newly diagnosed and were treatment naïve. Among the patients who have already been on treatment, the mean duration of treatment was 47 ± 47 months [range: 3–182]. FVC [forced vital capacity] was > 80% of the predicted values in 38% of the patients [*n* = 23], while 45% [*n* = 27] had FVC between 60 and 80% of the predicted values in the spirometry. Extrapulmonary involvement was present in 19 patients [31.7%], of which gastrointestinal system [*n* = 9] was the most commonly involved, followed by skin [*n* = 3] and eye [*n* = 2].

There was no statistically significant difference in the baseline clinical profile of the patients in the sarcoidosis group and the control group [Table 1].

**Table 1:** Baseline clinical profile

Clinical data	Sarcoid [*n* = 60]	Control [*n* = 60]	*P* value
Age	48 ± 9	49 ± 13	0.79
Sex			
Males	32 [53.3%]	37 [61.7%]	0.31
Females	28 [46.7%]	23 [38.3%]	
Diabetes mellitus	14 [23.3%]	19 [31.7%]	0.28
Systemic hypertension	12 [20%]	10 [16.7%]	0.67
Known coronary artery disease	1	0	0.32

### Electrocardiographic profile

Except for one patient in the sarcoidosis group, whose baseline ECG showed low atrial rhythm, all other patients in the sarcoid as well as the control groups were in sinus rhythm. The baseline electrocardiographic data are depicted in Table 2. The higher prevalence of intraventricular conduction defects [*n* = 41] in the study group was not statistically significant. Non-specific intraventricular conduction defects [IVCD] were common in both the groups, probably reflective of the age distribution and the underlying lung disease in both the groups. There was no difference in the pattern of IVCD in either group, in terms of the involvement of ECG leads.

**Table 2:** Electrocardiographic profile

Electrocardiographic data	Sarcoid [*n* = 60]	Control [*n* = 60]	*P* value
Rhythm			
Sinus rhythm	59	60	
Ectopic atrial rhythm	1	0	
PR interval [ms]	145 ± 22	138 ± 21	0.8
QRS duration [ms]	88 ± 15	86 ± 11	0.3
QT interval (corrected) [ms]	424 ± 34	431 ± 29	0.75
Conduction defect	41	34	0.23
Right bundle branch block	4	4	
Left bundle branch block	1	0	
Left anterior fascicular block	1	0	
Left posterior fascicular block	0	0	
Non-specific IVCD	35	30	
Non-specific IVCD			0.48
Inferior leads	18	18	
Anterior/lateral leads	1	2	
Diffuse	16	10	
Lead III			
Bifid P wave	9	2	0.029
Biphasic P wave	13	2	0.003
rsr’ pattern	3	5	0.115
Lead II bifid-P wave	10	4	0.09
Lead aVF-rsr’ pattern	5	0	0.03

*IVCD* intraventricular conduction defect

### Inferior wall leads [II,III,aVF]

A few salient electrocardiographic findings were observed in the limb leads representing the inferior wall [II,III,aVF], especially with regard to atrial depolarization.

### Biphasic P [Fig. 1]

In the sarcoid group, 13 patients had a biphasic P wave in lead III, which was found in only 2 patients in the control group [*P* < 0.003]. Among these, 4 patients had biphasic P wave in lead aVF as well. However, none had a biphasic P in lead II. The P wave duration in lead III was less than 120 ms in all these patients. The duration of terminal portion of P wave was less than 40 ms.

**Fig. 1:**
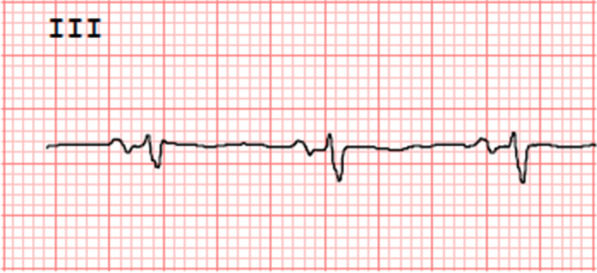
Typical biphasic P waves in lead III

### Bifid P

**Fig. 2:**
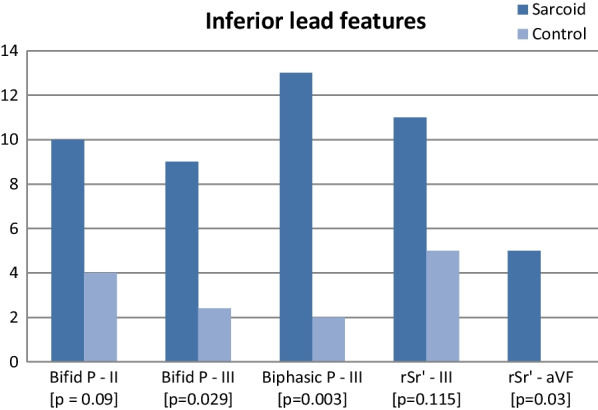
Salient findings in leads II,III,aVF

Similarly, bifid P wave in lead III was more common in the sarcoid group as compared to the controls [*P* = 0.029]. Among the 9 patients with a bifid P in lead III, 2 patients had other electrocardiographic features of left atrial abnormality including bifid P wave in lead II. However, this finding was not observed in lead aVF (Fig. 2).

Although bifid P wave in lead II was more prevalent in the study group overall, there was no statistically significant difference between the two groups [*P* = 0.09]. Half of these patients did not have other ECG features of left atrial enlargement [LAE] and none had echocardiographic LAE.

### rsr’

An rsr’ pattern was observed to be common in leads III and aVF in the sarcoid group, whereas rsr’ in aVF was not seen in the control group [*P* = 0.03].

### Clinical profile and ECG findings

Statistical analysis revealed that there was no correlation between any of the ECG findings and the clinical or spirometric profile of the patients (see Additional file 1). Neither the extent or severity of pulmonary disease, nor the presence of extrapulmonary involvement correlated with any of the electrocardiographic parameters observed. Review of follow-up data at 5 years revealed no evidence for cardiac sarcoidosis in any of these patients.

## Discussion

Cardiac sarcoidosis, which is often clinically silent, has been reported to be associated with an attributable mortality rate of up to 50 to 85% in autopsy series [18]. Most of these deaths are due to arrhythmias or conduction defects [13]. Since sudden death is often the presenting feature of cardiac sarcoidosis, early recognition of subclinical disease assumes utmost significance. The reported incidence of abnormal ECG in patients with clinically silent CS is only 3–8% in various studies [14, 15, 19, 20].

Many of the commonly described ECG findings in sarcoidosis such as RBBB are non-specific and are common in patients with other forms of pulmonary disease as well [21, 22]. Since abnormal ECG is regarded as an indication for screening for cardiac sarcoidosis [17], the early recognition of subtle abnormalities may facilitate timely referral of asymptomatic patients for advanced screening.

Our study, which compared the ECG findings in pulmonary sarcoidosis with an age-matched control group, has identified statistically significant prevalence of a few salient ECG abnormalities in patients with sarcoidosis. The salient findings observed in our study were as follows:

1. **Atrial depolarization abnormalities**
  Atrial depolarization abnormalities have not been described in the literature among the ECG findings in sarcoidosis [17, 30]. In our study, there was a greater prevalence of P wave abnormalities in lead III. Biphasic P wave was found in thirteen patients, and bifid P wave in nine, while both biphasic and bifid P waves in lead III were found in two. Thus a total of 20 patients had atleast one of these peculiar P wave features in lead III.
  [A] **Biphasic P waves** in inferior leads are usually a manifestation of delayed conduction of Bachmann's bundle [23, 24], which results in delayed activation of the left atrium. The impulse propagation from the lower right atrium to the left atrium in a caudo-cranial direction accounts for the biphasicity [25]. The greater prevalence of this finding in patients with pulmonary sarcoidosis may be an indication of early involvement of the atrial septum.
    Various studies have reported a higher incidence of supraventricular arrhythmias in patients with biphasic P wave in the inferior leads [26, 27]. Since cardiac sarcoidosis is associated with an increased incidence of atrial arrhythmias, including atrial fibrillation [7, 28], the greater prevalence of biphasic P wave may be a harbinger of atrial arrhythmias in sarcoidosis. The more vertical orientation of lead III as compared to the other inferior leads could explain the early appearance of biphasic P waves in this lead.
  [B] **Bifid P waves**: Bifid P wave in lead II is classically described as a sign of left atrial abnormality. Majority of patients with bifid P wave in lead III in our study had no other ECG or echocardiographic evidence to suggest left atrial abnormality or enlargement. Moreover, a bifid P wave was not present in lead II in most of these patients. There was no suggestion of left ventricular hypertrophy either. These P wave abnormalities could represent direct involvement of the atria by the underlying pathology, although confirmation with advanced imaging modalities like CMR or PET is desirable before drawing any conclusion in this regard.
2. **Ventricular depolarization abnormalities**
  Ventricular depolarization abnormalities such as slurring of QRS complexes, bundle branch blocks [right more than left], and increased duration of QRS complex have been reported to be more prevalent in those who were eventually detected to have cardiac sarcoidosis [22]. This could probably be explained by the relatively high prevalence of these findings in the age groups studied, as well as in those with underlying lung diseases. In our study, the higher prevalence of intraventricular conduction defects in the sarcoidosis group did not achieve statistical significance [67.1% vs. 56.6%, *P* = 0.23]. RBBB was equally prevalent in both the groups.
  Among the other abnormalities of the QRS complexes, our study demonstrated a greater prevalence of rsr’ pattern in leads III and aVF in the sarcoidosis group, of which rsr’ in lead aVF was found to be of statistical significance. All the aforementioned QRS abnormalities reflect the same underlying mechanism, namely an interruption in the progress of the electrical impulse through the myocardium, or abnormal fiber to fiber activation, which in the setting of sarcoidosis could be secondary to granulomas, scarring, or fibrosis [29].

### Correlation with severity of lung disease

There was no significant correlation of any of the ECG findings with the clinical or spirometric severity of pulmonary sarcoidosis or the multisystem involvement of the disease [the ECG findings and the corresponding indices of the lung disease are depicted in additional file 1]. It is to be noted that majority of the patients included in the study had either normal or mildly impaired lung function. The lack of correlation is not surprising, as cardiac involvement can occur at any stage of sarcoidosis, irrespective of the degree of pulmonary or systemic involvement [13, 30]. None of the patients in our study had cardiac manifestations of sarcoidosis at 5-year follow-up. Although this may be construed as a pointer against the pathologic significance of the ECG findings, it needs to be remembered that all these patients were undergoing therapy which could have modified the natural history of the disease.

The ECG findings widely described in subclinical sarcoidosis are abnormalities of ventricular depolarization. However, our study demonstrates a statistically significant prevalence of atrial depolarization abnormalities in patients with pulmonary sarcoidosis. Prompt recognition of subtle ECG abnormalities, correlation with imaging studies, and close clinical follow-up of the patients may aid in establishing the pathophysiologic relevance of these novel findings.

### Limitations

Our study is limited by the relatively smaller sample size and the observational study design. Correlation of ECG findings with advanced imaging would have been desirable to confirm the anatomic basis of these electrophysiologic abnormalities. However, the significantly higher prevalence of atrial depolarization abnormalities in the sarcoidosis group and the congruity of the ECG findings with the known atrial distribution of cardiac lesions in sarcoidosis, raise the possibility of subclinical cardiac involvement by the same pathology, which warrants further research.

## Conclusions

Our study demonstrates a greater prevalence of subtle ECG abnormalities in patients with pulmonary sarcoidosis as compared to patients with other forms of pulmonary disease. Atrial depolarization abnormalities such as biphasic P wave and bifid P wave in lead III were found to be more prevalent in patients with sarcoidosis. There was no correlation of these findings with the severity of pulmonary disease.

## Supplementary Information

**Additional file 1**. Appendix : Table showing the prevalence of various ECG abnormalities in varying degrees of pulmonary abnormalities.

## Data Availability

The datasets used and/or analyzed during the current study are available from the corresponding author on reasonable request.

## Abbreviations

ECG: Electrocardiogram
IVCD: Intraventricular conduction defects
MRI: Magnetic resonance imaging
PET: Positron emission tomography
CS: Cardiac sarcoidosis
VT: Ventricular tachycardia
