# Supplementary material for: Atrial depolarization abnormalities in pulmonary sarcoidosis
Source: Egypt Heart J. 2022 Oct 8;74:74. doi: 10.1186/s43044-022-00312-7 (PMC9547766; doi:10.1186/s43044-022-00312-7)
Supplement: Supplementary file 1 — Additional file 1. Appendix : Table showing the prevalence of various ECG abnormalities in varying degrees of pulmonary abnormalities. [file 43044_2022_312_MOESM1_ESM.docx]

|  | Bifid P-II,III | Biphasic P-III | rsr’-III | rsr’-aVF |
| --- | --- | --- | --- | --- |
| FVC <80% | 14% [p - 0.73] | 22% [p - 0.6] | 19% [p - 0.34] | 8.3% [p – 0.49] |
| DLCO >80% | 17% [p - 0.5] | 14% [p - 0.5] | 21% [p - 0.43] | 3.4% [p – 0.88] |
| Siltzbach stage  1  2  3  4 | 0  14 [p - 0.33]  40  12.5 | 20  21 [p - 0.9]  20  25 | 40  19 [p - 0.33]  20  0 | 0  12 [p – 0.52]  0  0 |

Appendix : Table showing the prevalence of various ECG abnormalities in varying degrees of pulmonary abnormalities
